# Differential Impact of Random GC Tetrad Binding and Chromatin Events on Transcriptional Inhibition by Olivomycin A

**DOI:** 10.3390/ijms23168871

**Published:** 2022-08-09

**Authors:** Alexandra K. Isagulieva, Dmitry N. Kaluzhny, Artemy D. Beniaminov, Nataliya V. Soshnikova, Alexander A. Shtil

**Affiliations:** 1 Institute of Gene Biology, Russian Academy of Sciences, 34/5 Vavilov Street, 119991 Moscow, Russia; 2 Gause Institute of New Antibiotics, 11 B. Pirogovskaya Street, 119021 Moscow, Russia; 3 Engelhardt Institute of Molecular Biology, Russian Academy of Sciences, 32 Vavilov Street, 119991 Moscow, Russia; 4 Blokhin National Medical Research Center of Oncology, 24 Kashirskoye Shosse, 115478 Moscow, Russia

**Keywords:** aureolic-acid-derived antibiotics, olivomycin A, RNA polymerase II, chromatin, transcription, gene expression

## Abstract

Olivomycin A (OA), an antibiotic of the aureolic acid family, interferes with gene transcription upon forming complexes with GC-rich regions in the DNA minor groove. We demonstrate that the mechanism of transcriptional deregulation is not limited to OA interaction with GC-containing binding sites for transcription factors. Using electrophoretic mobility shift assays and DNAse I footprinting of cytomegalovirus (CMV) promoter fragments carrying OA-preferred GC tetrads (CMVwt), we showed OA binding specifically to GC islands. Replacement of G for A in these tetrads (CMVmut) abrogated OA binding. Furthermore, OA decreased RNA polymerase II (RNAPII) binding to the CMVwt promoter and inhibited the reporter gene expression. In line with the absence of OA binding sites in CMVmut DNA, the expression driven from this promoter was weakly sensitive to OA. In the endogenous genes OA decreased RNAPII on promoters and coding regions. In certain cases this phenomenon was concomitant with the increased histone 3 abundance. However, the sensitivity to OA did not correlate with GC patterns around transcription start sites, suggesting that certain GC stretches play unequal roles in OA-induced transcriptional perturbations. Thus, OA affects transcription via complex mechanisms in which GC tetranucleotide binding causes RNAPII/chromatin alterations differentially manifested in individual gene contexts.

## 1. Introduction

Gene transcription, a fundamental process in all living organisms, is an attractive target for the treatment of cancer and other diseases [1,2,3]. Pharmacological inhibition of transcription, decades ago pioneered by actinomycin D [4], remains part of therapeutic regimens in patients with soft-tissue malignancies [5]. A plethora of novel transcriptional inhibitors of different chemical classes is being investigated in preclinical trials [6]. One class is a family of aureolic acid (AA) antibiotics produced by various strains of actinomycetes includes mithramycin, chromomycin A3, and olivomycin A (OA). These agents have been investigated as drug candidates due to a remarkable cytotoxic potency attributed to interference with global gene transcription [7]. Biosynthesis of mithramycin, chromomycin A3, and their derivatives has been investigated in detail [8]. In addition to their antiproliferative potency, AA antibiotics demonstrate an antiviral activity [9,10]. Mithramycin has been tested as a neuroprotector for Huntington’s disease and neuronal death [11,12].

Mechanistic studies proved that AA-derived antibiotics demonstrate a strong preference for GC-rich DNA sequences [13,14]. These compounds bind as dimers to the minor groove of the DNA double helix and form stable complexes with three (mithramycin [15]) or four (chromomycin A3 [16] and olivomycin A [17]) consecutive G/C base pairs. Conformational changes upon drug–duplex complex formation are thought to be responsible for alterations of the transcriptional machinery. Recent in vitro experiments have demonstrated that OA has similar binding affinity to different G/C tetranucleotide stretches although the rate of dissociation of OA–DNA complexes differs depending on specific G/C sequences. In the cell-free system, the GC context influenced the effects of OA on the traverse of T7 RNA polymerase along the DNA template [17]. In cell-based studies the AA family of antibiotics was investigated as DNA ligands capable of modulating the function of GC-preferred transcription factors. Ewing sarcoma is characterized by the t(11;22) (q24;q12) translocation that generates the Ewing sarcoma breakpoint region 1 and Friend leukemia virus integration 1 (EWS-FLI1) fusion transcription factor. Using high throughput mRNA analysis, Grohar et al. screened 50,000 small-molecular-weight compounds for the ability to inhibit EWS-FLI1-driven reporter gene transcription. In this series mithramycin showed the best (at nanomolar concentrations) inhibitory potency that paralleled its antitumor efficacy in vivo against xenografts [18]. EWS-FLI1 preferentially binds purine-rich microsatellite sequences that contain >9 5′-GGAA/T-3′ repeats [19]. Furthermore, Sp1 transcription factor binds to a GC-rich sequence (generalized formula 5’-(G/T)GGGCGG(G/A)(G/A)(C/T)-3’). In the cell-free system, mithramycin displaced Sp1 from its binding sites and protected *c*-*Myc* promoter from DNAse I [20]. AA antibiotics recruited to Sp1-activated genes disturb transcription [21,22] and decrease the amounts of Sp1 protein [23]. These data substantiate the perspective of AA antibiotics as antitumor drug candidates with the definitive molecular target, that is, GC specificity.

Although the details of OA–DNA binding have been studied largely in cell-free systems, the role of the cellular context, that is, regulation of individual gene expression including the chromatin level, remains less understood. Several studies demonstrated that incubation of tumor cells with AA antibiotics changed the balance of acetylation/methylation of DNA and histones [24,25,26]. Furthermore, binding of AA antibiotics to the minor groove of DNA can induce dissociation of the chromatin-remodeling complex from DNA and inhibit the activity of Dnmt3a methyltransferase [27,28]. Additionally, the affinity of AA antibiotics to DNA is significantly affected by nucleosome density because these agents and N-terminal tails of histone 3 compete for binding to the minor groove [29,30]. These findings indicate that drug–duplex interaction, definitely the primary event causatively associated with the efficacy of AA antibiotics, has many consequences, therefore a simplified model of blocked RNA polymerase II (RNAPII) movement may be insufficient. In particular, do AA antibiotics modulate gene transcription by binding to randomly located GC tetrads or specifically to the consensus binding sites for GC-preferred transcription factors?

In this study we analyzed OA binding to the chimeric DNA construct in vitro and the effects of this interaction in the cellular context. We found that steady-state mRNA levels as well as distribution of RNAPII, histone H3, and BRG1 ATPase of SWI/SNF remodeling complex on the promoters and coding DNA regions varied in endogenous genes differentially sensitive to OA. Our findings demonstrated that, although GC tetramers are the critical targets for transcriptional deregulation by OA in the cell-free system, the roles of individual tetrads in the endogenous genes differed, and so did the molecular events mechanistically associated with OA–DNA complex formation.

## 2. Results and Discussion

### 2.1. GC Tetrads in the Cytomegalovirus Promoter Are Critical for OA–DNA Complex Formation: An In Vitro Study

**Figure 1:**
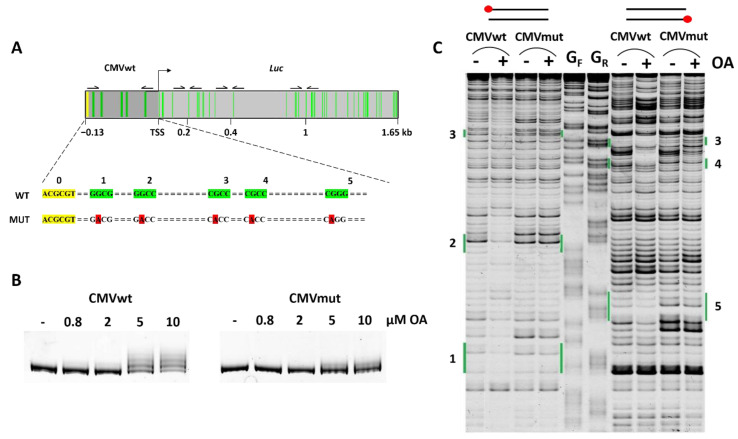
GC tetrads as sites of OA–DNA complex formation. (**A**) Tentative OA binding sites in minimal CMVwt promoters are marked in yellow and green. The yellow site used for cloning remained intact whereas in sites 1–5 (green) guanine residues were substituted for adenine (red). (**B**) EMSA of OA complexes with CMVwt and CMVmut DNA fragments. Note the more slowly migrating DNAs after incubation of CMVwt fragment with 5 µM or 10 µM OA. (**C**) DNase I probing of CMVwt and CMVmut fragments. Forward (left) or reverse (right) strands were 5′-end labeled with FAM (red circles). G_F_ and G_R_ are guanine sequencing lanes for the sense and complementary strands, respectively, obtained by treatment of DNA fragments with dimethylsulfate (see text and Section 3 for details). Positions of tetranucleotides 1–5 are shown in green as in (**A**).

GC tetrads with specific positions of G/C nucleotides have been shown to be critical for OA–DNA complex formation in vitro [17]. These short DNA repeats in gene promoters are responsible for binding of cognate transcription factors such as Sp1 and others [31,32]. However, Sp1 binding site(s) in the promoter may not be necessary for transcriptional intervention in the cellular context because OA suppressed transcription of the gene driven by the promoter that lacked Sp1 binding sites [33]. To investigate whether GC tetrads beyond transcription-factor binding sites are critical for OA mediated transcriptional inhibition we constructed two vectors with minimal CMV promoter. As shown in Figure 1A, this promoter contains GC tetrads with individual nucleotide residues at different positions (green boxes 1–5; see also Supplementary Materials, Schemes S1 and S2 for full DNA sequence), the potential OA binding sites. No Sp1 consensus binding sites were present in this promoter. We cloned the full-length luciferase (*Luc*) gene under the wild type (CMVwt) or mutant (CMVmut) CMV promoter. In the latter construct, each of tetrads 1–5 was mutagenized by replacing G for A (Figure 1A). Substitution of the guanine residue in central GC base pairs is expected to completely abrogate the binding of the antibiotic since the amino group of guanine is absolutely required for interaction with the aglycon moieties in the OA dimer [17]. The restriction site MluI at the 5′ end of the construct remained intact (yellow square in Figure 1A).

To test whether OA is able to bind tetrads 1–5 we used electrophoretic mobility shift assay (EMSA) and DNase I footprinting. For OA–DNA binding assays, a 144 bp fragment amplified from CMVwt or CMVmut DNA was incubated with OA and resolved by electrophoresis in 8% non-denaturing polyacrylamide gel. At <2 µM OA CMVwt, fragments migrated as one single band (Figure 1B). With bigger OA concentrations we detected additional slowly migrating bands attributable to DNA complexes with one or more OA dimers. In contrast, fragments amplified from CMVmut demonstrated only a slight shift of in-gel mobility due to the complex formed by OA with the sole non-mutated site (Figure 1A).

To substantiate the role of specific GC tetrads in OA–DNA complex formation, we identified DNA stretches protected from digestion by DNase I. We used PCR-based fluorescent labeling of each strand that allowed for a reliable reading of the footprint from both ends of the fragment (Figure 1C). The addition of OA to CMVwt fragments resulted in the protection of individual regions from cleavage by DNAse I. This effect was not observed in CMVmut fragments where the patterns of DNase I cleavage in the presence and absence of OA were identical (Figure 1C, green bars).

Sites of CMVwt DNA fragments protected by the antibiotic may not exactly coincide with putative GC tetranucleotide sites. Moreover, the degree of protection of individual sites is different. DNase I seems an ideal probe for OA footprinting because this enzyme also interacts with the double helix in the minor groove. However, the enzymatic efficacy of DNAse I is sensitive to the geometry of the double helix. AA antibiotics are known to bend and disturb the structure of the DNA duplex, therefore their binding may distort the double helix even at considerable distances from the site of enzyme–DNA interaction [34,35].

### 2.2. OA–DNA Complex Formation Impedes Transcription Driven by CMVwt but Not Cmvmut

**Figure 2:**
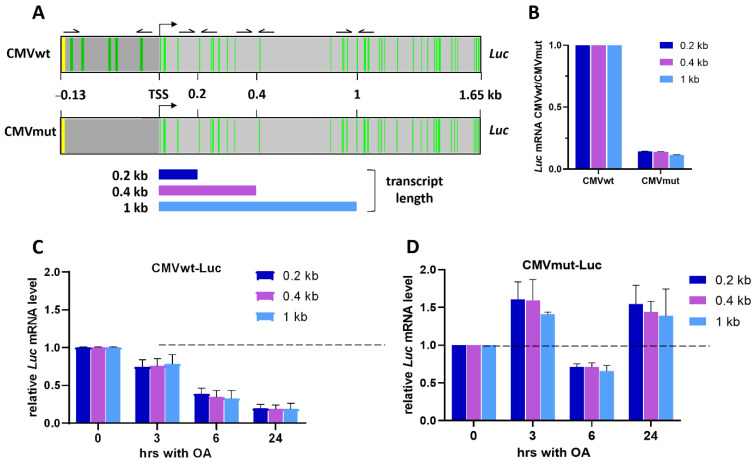
Effects of OA on transcription depend on GC tetrads in the promoter. (**A**) Schematic representation of GC tetrads in CMV-Luc constructs. Green stripes in the CMVwt construct represent SGSS and SSCS tetrads (S stands for G or C). These sites were mutated in the CMVmut-Luc promoter (no green strips). Arrows are the same as in Figure 1A. (**B**) Basal *Luc* transcription depends on intact GC tetrads in the promoter. Expression of CMVmut was taken as 1, expression of CMVmut-Luc normalized to CMVwt-Luc. (**C**,**D**) Time-course of relative abundance of *Luc* transcripts (normalized to mRNA of *RPLP0*). Experiments were performed twice, each measurement in triplicate.

With the knowledge about in vitro OA–DNA binding sites in the promoter-*Luc* constructs, we next used the cellular context to study the role of GC tetrads as targets for transcriptional deregulation by OA. CMVwt-Luc or CMVmut-Luc constructs (Figure 2A) were introduced into HEK293T cells followed by selection of stable transfectants with puromycin (HEK293T-CMVwt-Luc and HEK293T-CMVmut-Luc sublines). The expression of *Luc* transcripts driven from the respective CMV-derived promoter was determined at different distances from TSS and normalized on the abundance of *RPLP0* mRNA. Levels of *Luc* mRNA driven from CMVwt promoter were taken as 1. Importantly, basal *Luc* expression from the CMVwt construct was ~7–8 times more pronounced compared to CMVmut-Luc (Figure 2B), further substantiating the critical role of promoter GC islands in transcription.

The HEK293T-CMVwt-Luc and HEK293T-CMVmut-Luc cells were exposed to 100 nM OA for 1–24 h. Amounts of *Luc* mRNA transcribed from CMVwt promoter decreased in a time-dependent manner, with a significant decrease by 24 h (>30% compared to untreated control; Figure 2C). In contrast, the abundance of *Luc* mRNA in HEK293-CMVmut-Luc cells did not decrease gradually but varied over time (Figure 2D). In each subline the 0.2 kb, 0.4 kb, and 1 kb transcripts changed coordinately, that is, OA caused no preferential deregulation of the shorter vs. longer mRNAs. These results indicated that, in the absence of GC tetrads in the promoter, OA did not efficiently interfere with transcription regardless of the presence of GC tetrads in CDS.

### 2.3. Reversibility of OA-Induced Transcriptional Attenuation

**Figure 3:**
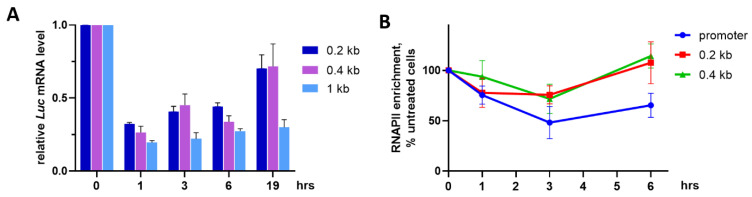
Time- and size-dependent restoration of transcripts after OA washing. HEK293T-CMVwt-Luc cells were treated 100 nM OA for 3 h, washed with saline, and incubated in OA-free medium for indicated time intervals. (**A**) qRT-PCR analysis of *Luc* mRNAs of different length. Values (normalized to *RPLP0* signal) are relative to those prior to OA washing (0 h). (**B**) Abundance of total RNAPII on the *Luc* gene (ChIP assays).

Because AA-derived antibiotics bind DNA non-covalently, their effects may be reversible, as has been shown for mithramycin [19]. The initial decrease of *Luc* mRNA abundance in HEK293T-CMVwt-Luc cells by 3 h with OA (Figure 2C) continued after drug removal and incubation in the antibiotic-free medium for 1 h (Figure 3A). By 1 h in OA-free medium, relative abundances of 0.2 kb and 0.4 kb transcripts were ~30% of respective values before OA removal (100 %). During the course of incubation in the drug-free medium mRNAs gradually increased with time, reaching ~70% of the level before OA removal by 19 h (Figure 3A). In contrast, the longer (1 kb) transcripts were significantly repressed: the initial decrease to ~20% at 1 h remained consistently low for up to 19 h in the drug-free medium (<30%). These data indicated that, after OA washing, transcription resumed in an asynchronous manner. Re-appearance of long mRNAs was delayed compared to shorter transcripts. One may hypothesize that GC tetrads in the promoter or gene body are functionally non-equivalent, otherwise, posttranscriptional regulation (i.e., message stability) cannot be excluded. 

In line with qRT-PCR data, the chromatin immunoprecipitation (ChIP) analysis showed time-dependent changes of the presence of total RNA polymerase II (RNAPII) on the promoter and 3′ *Luc* gene regions proximal to TSS. Incubation of HEK293T-CMVwt-Luc cells with 100 nM OA for 3 h decreased RNAPII presence on 0.2 kb and 0.4 kb DNAs. This decrease was sustained within 3 h after OA washing followed by gradual restoration of RNAPII by 6 h on each of studied gene regions (Figure 3B). Thus, the effects of OA on RNAPII abundance were directly associated with the presence of the antibiotic in the culture medium. However, the rate of transcriptional recovery along the gene can be different given that newly detectable RNAPII molecules on the promoter retarded compared to CDS regions. In turn, this difference might be attributed to an unequal functional significance of GC tetrads in individual gene loci.

### 2.4. Differential Effects of OA on Gene Occupancy by Total RNAPII

**Figure 4:**
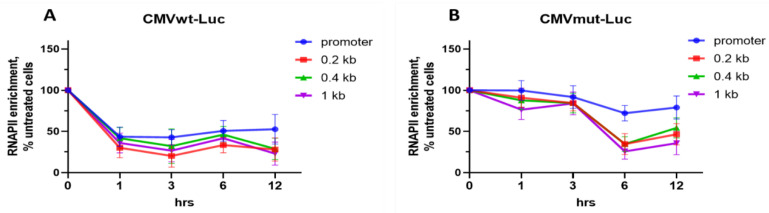
OA modulates the abundance of molecules depending on promoter GC context. HEK293T-CMVwt-Luc (**A**) or HEK293T-CMVmut-Luc (**B**) cells were treated with 100 nM OA for indicated time intervals followed by ChIP assays (see Section 3). Shown are enrichments in total RNAPII molecules on the *Luc* promoter and on CDS. RNAPII enrichment in untreated samples (0 h, input) was taken as 100%. Data are mean ± SD of two independent experiments, each value measured in triplicate.

Next, we investigated whether transcriptional deregulation by OA involves an altered presence of RNAPII molecules along the gene, and whether these effects depend on the GC context of the promoter. ChIP assays were used to compare the abundance of total RNAPII in OA-treated HEK293T-CMVwt-Luc or HEK293T-CMVmut-Luc cells. Amounts of total RNAPII on the CMVwt promoter and *Luc* CDS had decreased within 1 h (Figure 4A). This effect remained sustained for 12 h, in line with the time-dependent decrease of *Luc* mRNA. On the contrary, the abundance of RNAPII on the CMVmut promoter did not change markedly during the course of OA treatment (Figure 4B). These results strongly suggested that the formation of complexes of OA and GC tetrads on the CMVwt promoter is detrimental for transcription whereas the absence of sites for strong complexes in the CMVmut promoter alleviates the inhibitory effect of OA. Nevertheless, GC tetrads in the *Luc* CDS remained intact; accordingly, with OA, in HEK293T-CMVmut-Luc cells, RNAPII molecules on *Luc* CDS decreased by 6 h (Figure 4B). One may hypothesize that OA interaction with GC tetrads in the *Luc* gene body provides conformational hindrances for polymerases that reached these sites. Thus, GC tetrads in the promoter, or, to a lesser extent in the gene body, are major sites for OA to interfere with transcription.

### 2.5. Does the GC Context Matter?

**Figure 5:**
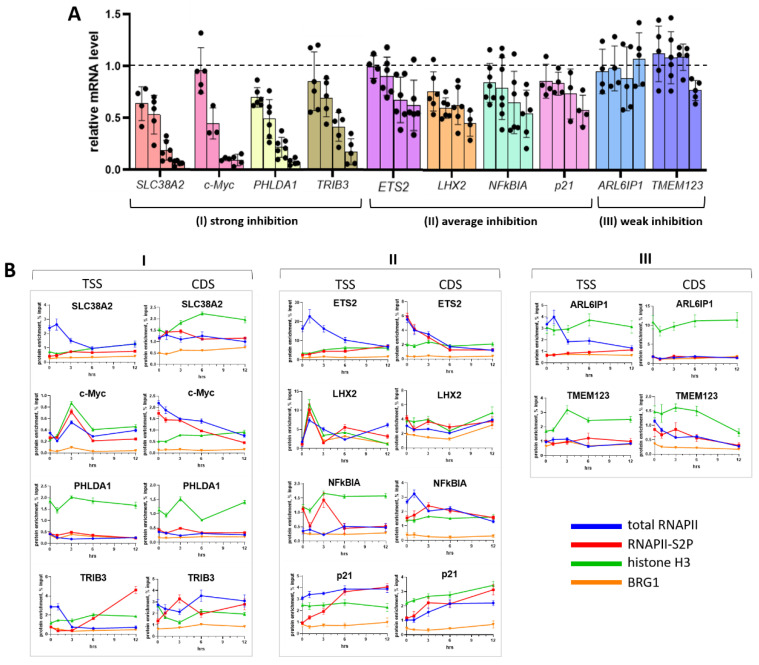
Differential gene sensitivity to the inhibitory effect of OA. (**A**) HCT116 cells were incubated with 100 nM OA for up to 12 h. Relative mRNA levels were determined by qPCR and normalized to *RPLP0* mRNA. Signals in untreated cells (0 h) were taken as 1 (dotted line). In each group of four bars (from left to right): 1 h, 3 h, 6 h, and 12 h, respectively. (**B**) Time-dependent changes of total RNAPII, elongating RNAPII-S2P, core histone H3, and BRG1 around TSS or on CDS. Data are mean ± SD of three measurements. Genes were grouped according to their sensitivity to OA: highly sensitive genes (I, left panel), averagely sensitive genes (II, middle panel), and weakly sensitive genes (III, right panel).

Effects of AA derivatives may not be confined to transcriptional inhibition of endogenous genes. Based on previous studies [21,36,37] we selected a set of genes known to be markedly deregulated by AA-family antibiotics, namely, genes encoding transmembrane proteins (*SLC38A2, ARL6IP1,* and *TMEM123*), transcription factors (*c-Myc, PHLDA1, ETS2,* and *LHX2*), and the NFkB pathway (*NFkBIA* and *TRIB3*) to analyze transcriptional responses to OA. The HCT116 human colon carcinoma cell line served as a test system for consistency with previous reports [37,38]. Cells were incubated with 100 nM OA for 1–12 h and processed for qRT-PCR and ChIP assays. Genes fell into three groups according to their response to OA: (I) highly sensitive (mRNAs decreased two-fold within 1–6 h and continued to decrease until 12 h: *SLC38A2, PHLDA1, c-Myc,* and *TRIB3*), (II) averagely sensitive (mRNAs decreased by ~50% by 12 h: *ETS2, LHX2, NFkBIA,* and *p21*), and (III) weakly sensitive (mRNAs changed insignificantly: *ARL6IP1* and *TMEM123*) (Figure 5A).

Next, we used ChIP [39] to analyze whether the OA-altered expression of individual genes is associated with the abundance of total and elongating (phosphorylated at Ser-2; S2P [40]) RNAPII molecules, the core histone H3 and BRG1 subunit of the SWI/SNF chromatin remodeling complex. In all genes except *PHLDA1, p21*, and *LHX2*, treatment with 100 nM OA for 12 h caused a decrease of total RNAPII levels near TSS and/or on CDS (Figure 5B, blue curves). In the *ETS2, c-Myc, LHX2, NFκBIA*, and *ARL6IP1* genes this effect was preceded by a sharp increase of total RNAPII detectable as early as after 1–3 h. Amounts of elongating RNAPII-S2P in some genes did not change or even increased around TSS and on CDS of *ARL6IP1, TRIB3, ETS2*, and *p21* genes (Figure 5B, red curves). 

Noticeable changes were also detectable with the core histone H3. In the *c-Myc, LHX2, p21, SLC38A2, NFκBIA, ETS2*, and *TMEM123* genes, treatment with OA was associated with a statistically significant increase of histone H3 around TSS or on CDS (Figure 5B, green curves). This important observation, namely, induction of nucleosomal occupancy in the course of gene inhibition, reflects a previously unattended mechanism of transcriptional deregulation by OA. AA antibiotics have been shown to compete with the N-terminal domain of histone H3 for minor DNA groove-binding [29,30]. In this regard, it is interesting to reveal how DNA-associated OA can regulate the nucleosome occupancy.

Compared to RNAPII and histone H3, treatment with OA weakly changed BRG1. However, on the *c-Myc* and *LHX2* genes, BRG1 accumulated in the vicinity of TSS within 1–3 h. The presence of histone H3 increased along with BRG1 albeit to a different extent: 13-fold on the *LHX2* gene vs. 2-fold on the *c-Myc* gene. Interestingly, by 6 h *c-Myc* mRNA decreased by >50% whereas *LHX2* mRNA changed moderately (Figure 5A). One may hypothesize that, despite the increased density of nucleosomes near the promoter, chromatin remodeling on *LHX2* can proceed more efficiently than on *c-Myc*. Alternatively, or in addition, BRG1 can act as a transcriptional repressor [41]. Posttranscriptional modifications are also important since *c-Myc* mRNA is known to be short-lived [42].

### 2.6. Sensitivity of Individual Genes to OA Is Independent of GC Tetrad Location

**Figure 6:**
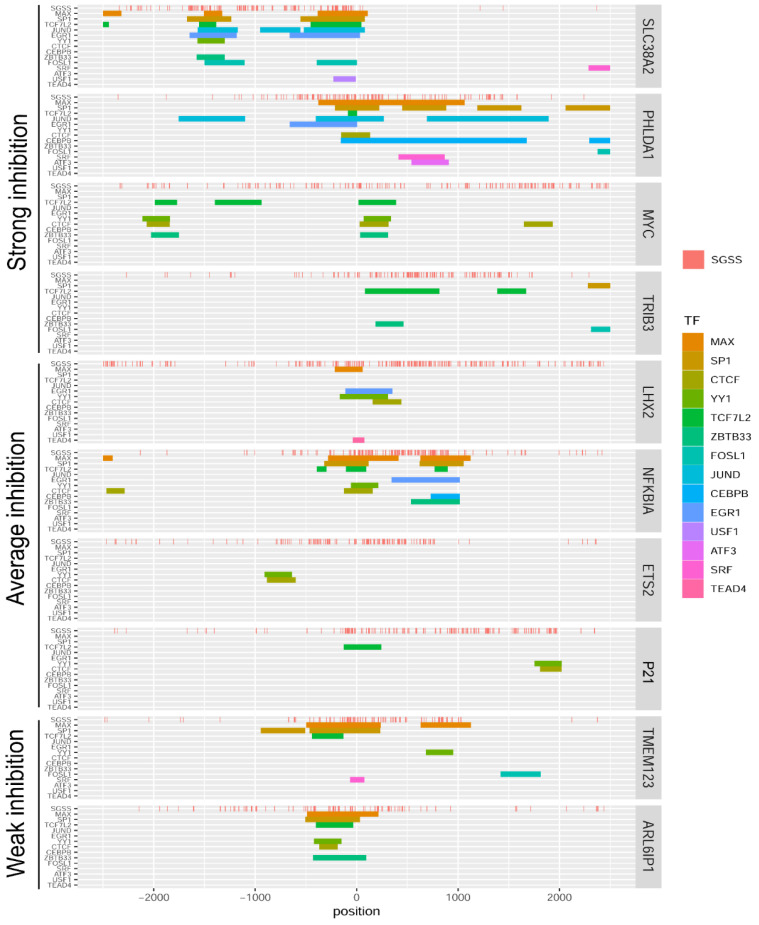
Mapping of GC tetrad distribution (SGSS, top red stripes in each gene block) and location of transcription-factor (TF) binding sites (colored). The analysis was based on the Encode database (see Section 3). Genes were grouped according to their transcriptional sensitivity to OA.

According to previous results [17], we mapped GC tetrads as tentative OA binding sites in the region −2500 to +2500 bp around TSS. No dependence between the presence or distribution of GC tetrads and the sensitivity to OA was revealed (Figure 6, the top panel SGSS in each gene block). Indeed, the most ‘irresponsive’ genes, *ARL6IP1* and *TMEM123*, contained several GC tetrads close to TSS. In contrast, in the *SLC38A2* gene, only a few GC tetrads were found 5′ from TSS whereas OA strongly inhibited the expression of this gene (Figure 5A). Moreover, even within the group of highly sensitive genes the number and localization of putative OA binding sites differed significantly. Although the analysis of a bigger set of genes is worthy, the present data argue against direct relationship between the number of OA binding sites and the effects on transcription. 

Interference with transcription factors for binding to the duplex can be attributable for transcriptional deregulation by OA. To identify transcription factors in addition to the canonical Sp1, we analyzed the regions from −2500 bp to +2500 bp from TSS of studied genes (Figure 6, colored stripes). The Encode database (https://www.encodeproject.org/, accessed on 24 May 2022) contains 17 ChIPseq tracks of transcription factors for the HCT116 cell line, three of which (ELF1, REST, and ZNF274) were not found in the vicinity of TSS in the studied genes. Distribution of binding peaks of 14 transcription factors around TSS relative to potential sites of OA–DNA interaction (d(SGSS)) is shown in Figure 6. One may suggest that OA can compete with many transcription factors.

To get an initial insight into the involvement of individual transcription factors we analyzed the positions of their binding sites in genes that differentially responded to OA (Figure 5A and Figure 6). Binding peaks of Max, Sp1, CTCF, YY1, TCF7L2, ZBTB33, and FOSL1 were present in promoters of the majority of genes regardless of their sensitivity to OA. Peaks for JUND, CEBPB, EGR1, USF1, ATF3, SRF, and TEAD4 were found only in the genes whose expression was attenuated by OA suggesting that the latter factors and OA may compete for binding to DNA. However, our search was confined to a limited gene set; a reliable statistical analysis requires a genome-wide study.

## 3. Materials and Methods

### 3.1. Cell Lines and Treatment

The colon carcinoma HCT116 and human embryonic kidney HEK293T (both from American Type Culture Collection, Manassas, VA, USA) cell lines and the genetically modified sublines generated in this study were cultured in Dulbecco modified Eagle’s medium (PanEco, Moscow, Russia) supplemented with 10% fetal bovine serum (HyClone, Logan, UT, USA), 2 mM L-glutamine, 100 U/mL penicillin, and 100 µg/mL streptomycin (PanEco, Russia) at 37 °C, 5% CO_2_ in a humidified atmosphere. OA was produced at the pilot plant of Gause Institute of New Antibiotics, Moscow [43] and provided by Professor A.N. Tevyashova. The stock solution of OA in DMSO (10 mM) was stored at −20 °C. Serial dilutions in the culture medium were prepared immediately before experiments. 

### 3.2. Antibodies

The anti-BRG1 antibody was produced as described [44]. Antibodies against histone H3 (Ab18521) and RNAPII-S2P (Ab252855) were from Abcam (Cambridge, UK). Anti-RNAPII (#151247) was from Active Motif (Carlsbad, CA, USA). 

### 3.3. Plasmids, Cloning, and Cell Transfection

To create CMVwt-Luc reporter-expressing vector, minimal CMVwt promoter DNA was PCR amplified from pcDNA3.1 vector (ThermoFisher Scientific, Waltham, MA, USA) and cloned into pcDNA-3Fl vector. The full-length *Luc* gene DNA was cloned from pBV-Luc wt MBS1-4 (Plasmid #16564) into pcDNA-CMVwt-3Fl. Synthesis of CMVmut fragment and assembly of the CMVmut-Luc vector based on the existing CMVwt-Luc construct was made in Evrogen (Moscow, Russia). Vector DNA sequences are given in Schemes S1 and S2. HEK293T cells were plated in 6 cm Petri dishes. After reaching 70–80% confluence cells were transfected with the respective vector DNA (6 μg) using 12 μL polyethylenimine (Polysciences, Warrington, PA, USA) in 600 μL OptiMEM (ThermoFisher Scientific, Waltham, MA, USA) for 24 h, and washed with PBS. To select the cells with inserts puromycin (PanEco, Russia; 0.5 mg/mL) was added to the culture medium for 14 days. 

### 3.4. Real-Time qPCR

Total RNA from cultured cells was isolated using the ExtractRNA (Evrogen) according to the manufacturer’s manual. Coding DNA was obtained by reverse transcription of 1–2.5 μg of total RNA using the Revert Aid Kit (ThermoFisher Scientific, Waltham, MA, USA). Gene expression was evaluated by real-time qPCR (Roshe LightCycler, Basel, Switzerland) using EvaGreen dye (Biotium, Fremont, CA, USA). The *RPLP0* mRNA levels were used for signal normalization. Results were analyzed using delta-delta Ct method. The list of primers is given in Table S1. Experiments were carried out as at least two independent biological replicates, each reaction run in triplicate. Values are mean ± SD, statistical analysis was performed using GraphPad Prism 8.

### 3.5. Chromatin Immunoprecipitation

Immunoprecipitation of chromatin was performed essentially as described [45]. Briefly, 1 × 10^6^ cells per 10 cm Petri dishes were incubated with 100 nM OA, then cross-linked with 1% formaldehyde for 10 min, treated with by 2.5 M glycine, and washed in 3 × 1 mL saline supplemented with protease inhibitor cocktail (Sigma-Aldrich, Burlington, MA, USA). Chromatin was sonicated to produce ~500 bp fragments, samples were centrifuged (13,000× *g*, 4 °C 15 min) and incubated with bovine-serum-albumin-blocked MabSelect beads (GE Health, Waltham, MA, USA) and anti-RNAPII (1:5), anti-RNAPII-S2P (1:5), anti-H3 (1:5), or anti-BRG1 (1:2) antibodies overnight at 4 °C. Samples prior to immunoprecipitation were used as the input control. The precipitated beads were washed and the DNA of input and precipitated samples was eluted and extracted by the phenol–chloroform method. DNA fragments were amplified by PCR. The list of primers is given in Table S1. The input was adjusted according to the dilution factor. Protein enrichments were calculated as the percentage of the initial input (100%) calculated by the delta-delta Ct method. Experiments were conducted at least twice, each reaction run in triplicate.

### 3.6. EMSA

OA–DNA complexes were formed in the binding buffer (20 mM Tris-HCl pH 8.0, 50 mM KCl) with 500 nM of DNA fragments and indicated OA concentrations for 20 min at 25 °C. Samples were resolved in a 8% non-denaturing polyacrylamide gel electrophoresis and visualized on a Typhoon FLA 9500 fluorescence scanner (GE Healthcare, Chicago, IL, USA) equipped with a 473 nm blue LD laser and LBP filter.

### 3.7. DNAse I Protection Assays

Fragments (144 bp) of CMVwt and CMVmut promoter DNA were amplified from respective *Luc* vectors. Primer pairs FAM-cmvF/cmvR: 5′-FAM-GATATACGCGTCGAGGTA/GTACCAGGAGGCTGGAT-3′ or cmvF/FAM-cmvR: 5′-GATATACGCGTCGAGGTA/FAM-GTACCAGGAGGCTGGAT-3′ were used to generate 5′-end labeled fluorescent sense or antisense DNA strands, respectively. Complexes of OA (10 µM) with FAM-labeled CMVwt or CMVmut DNA fragments (500 nM each) were preincubated in the binding buffer supplemented with 2 mM MgCl_2_ at 25 °C for 20 min and then digested for 2 min with 0.03 units of DNase I (ThermoFisher Scientific). Reactions were stopped by the addition of ethanol. Guanine sequencing lanes were obtained by incubating DNA fragments in 2% dimethylsulfate at room temperature for 3 min followed by ethanol precipitation and incubation (30 min, 95 °C) in the loading buffer (90% formamide, 0.025% bromophenol blue, 0.025% xylene cyanol). The digested products were separated by electrophoresis in a denaturing 10% polyacrylamide gel containing 7M urea and visualized on a Typhoon FLA 9500 fluorescence scanner.

### 3.8. Mapping GC Tetrads and TF Consensus Binding Sites

Location of GC tetrads was analyzed using the code available at GitHub: https://github.com/uzhny/olivomycin-2022 (accessed on 25 July 2022). The code marked all SGSS type tetrads (S = G or C) in red [10]. The code mapped the binding sites of Max, Sp1, CTCF, YY1, TCF7L2, ZBTB33, FOSL1, JUND, CEBPB, EGR1, USF1, ATF3, SRF, and TEAD4 (Encode database).

### 3.9. Statistical Analysis

Experiments were carried out at least in two biological replicates, each measurement in triplicate. qRT-PCR data are presented as mean ± SD. Statistical analysis was performed using GraphPad Prism 8 and MO Excel software.

## 4. Conclusions

In summary, our results with genetically defined cell-free systems and in cell culture indicated the critical role of complex formation by OA and GC tetrads in transcriptional deregulation. The lack of correlation between the presence/positioning of binding sites for GC-preferred transcription factors and the responsiveness of individual genes to the antibiotic argues against the explanation of OA effects solely by competition with transcription factors for duplex availability. Randomly located GC tetranucleotides rather than the consensus sequences for GC-specific transcription factors appeared to be necessary for OA to exert its anti-transcriptional effect. These findings broaden the functional significance of OA–target interaction. 

However, the roles of certain GC tetrads vary. Although numerous GC tetrads across the genome can bind OA, we demonstrated the differential responsiveness of individual genes to the antibiotic. Importantly, epigenetic factors such as chromatin context emerged as the mechanisms of transcriptional inhibition by OA. Within the set of studied genes the chromatin events were present to a varying extent, adding another level of complexity to the mechanistic model. Altogether, binding to random GC tetrads is a necessary prerequisite for transcriptional deregulation by OA, this key event alone would cause local conformational hindrances for the traverse of the transcriptional complex. Other mechanisms such as chromatin changes (wherever relevant) and posttranscriptional modifications further determine the life-time of individual transcripts. 

## Data Availability

The GC tetrad location in vectors and gene sequences was analyzed using the code available at GitHub: https://github.com/uzhny/olivomycin-2022 (accessed on 25 July 2022).

## Supplementary Materials

The supporting information can be downloaded at: https://www.mdpi.com/article/10.3390/ijms23168871/s1.
