# Supplementary material for: Differential Impact of Random GC Tetrad Binding and Chromatin Events on Transcriptional Inhibition by Olivomycin A
Source: Int J Mol Sci. 2022 Aug 9;23(16):8871. doi: 10.3390/ijms23168871 (PMC9408465; doi:10.3390/ijms23168871)
Supplement: Supplementary file 1 [file ijms-23-08871-s001.zip › ijms-1815041-supplementary.pdf]

Supplement Materials

Scheme S1. Minimal CMVwt sequence.

ACGCGTCGAGGTAGGCGTGTACGGTGGGAGGCCTATATAAGCAGAGCTCGTTTAGTGAACCGTCAGATCGC
CTGGAGACGCCATCCACGCTGTTTTGACCTCCATAGAAGACACCGGGACCGATCCAGCCTCCTGGTACCATG
GAAGACGCCAAAAACATAAAGAAAGGCCCGGCGCCATTCTATCCGCTGGAAGATGGAACCGCTGGAGAGC
AACTGCATAAGGCTATGAAGAGATACGCCCTGGTTCCTGGAACAATTGCTTTTACAGATGCACATATCGAGG
TGGACATCACTTACGCTGAGTACTTCGAAATGTCCGTTCGGTTGGCAGAAGCTATGAAACGATATGGGCTGA
ATACAAATCACAGAATCGTCGTATGCAGTGAAAACTCTCTTCAATTCTTTATGCCGGTGTTGGGCGCGTTATT
TATCGGAGTTGCAGTTGCGCCCGCGAACGACATTTATAATGAACGTGAATTGCTCAACAGTATGGGCATTTC
GCAGCCTACCGTGGTGTTCGTTTCCAAAAAGGGGTTGCAAAAAATTTTGAACGTGCAAAAAAAGCTCCCAAT
CATCCAAAAAATTATTATCATGGATTCTAAAACGGATTACCAGGGATTTCAGTCGATGTACACGTTCGTCAC
ATCTCATCTACCTCCCGGTTTTAATGAATACGATTTTGTGCCAGAGTCCTTCGATAGGGACAAGACAATTGCA
CTGATCATGAACTCCTCTGGATCTACTGGTCTGCCTAAAGGTGTCGCTCTGCCTCATAGAACTGCCTGCGTGA
GATTCTCGCATGCCAGAGATCCTATTTTTGGCAATCAAATCATTCCGGATACTGCGATTTTAAGTGTTGTTCCA
TTCCATCACGGTTTTGGAATGTTTACTACACTCGGATATTTGATATGTGGATTTCGAGTCGTCTTAATGTATAG
ATTTGAAGAAGAGCTGTTTCTGAGGAGCCTTCAGGATTACAAGATTCAAAGTGCGCTGCTGGTGCCAACCCT
ATTCTCCTTCTTCGCCAAAAGCACTCTGATTGACAAATACGATTTATCTAATTTACACGAAATTGCTTCTGGTG
GCGCTCCCCTCTCTAAGGAAGTCGGGGAAGCGGTTGCCAAGAGGTTCCATCTGCCAGGTATCAGGCAAGGA
TATGGGCTCACTGAGACTACATCAGCTATTCTGATTACACCCGAGGGGGATGATAAACCGGGCGCGGTCGGT
AAAGTTGTTCCATTTTTTGAAGCGAAGGTTGTGGATCTGGATACCGGGAAAACGCTGGGCGTTAATCAAAGA
GGCGAACTGTGTGTGAGAGGTCCTATGATTATGTCCGGTTATGTAAACAATCCGGAAGCGACCAACGCCTTG
ATTGACAAGGATGGATGGCTACATTCTGGAGACATAGCTTACTGGGACGAAGACGAACACTTCTTCATCGTT
GACCGCCTGAAGTCTCTGATTAAGTACAAAGGCTATCAGGTGGCTCCCGCTGAATTGGAATCCATCTTGCTC
CAACACCCCAACATCTTCGACGCAGGTGTCGCAGGTCTTCCCGACGATGACGCCGGTGAACTTCCCGCCGCC
GTTGTTGTTTTGGAGCACGGAAAGACGATGACGGAAAAAGAGATCGTGGATTACGTCGCCAGTCAAGTAAC
AACCGCGAAAAAGTTGCGCGGAGGAGTTGTGTTTGTGGACGAAGTACCGAAAGGTCTTACCGGAAAACTCG
ACGCAAGAAAAATCAGAGAGATCCTCATAAAGGCCAAGAAGGGCGGAAAGATCGCCGTGTAAT

Color codes: yellow, restriction sites for MluI (left) and NcoI (right) used in ligation and cloning; green, OA putative OA binding sites; gray, *Luc* CDS.

Scheme S2. Minimal CMVmut sequence.

ACGCGTCGAGGTAGACGTGTACGGTGGGAGACCTATATAAGCAGAGCTCGTTTAGTGAACCGTCAGATCAC
CTGGAGACACCATCCACGCTGTTTTGACCTCCATAGAAGACACCAGGACCGATCCAGCCTCCTGGTACCATG
GAAGACGCCAAAAACATAAAGAAAGGCCCGGCGCCATTCTATCCGCTGGAAGATGGAACCGCTGGAGAGC
AACTGCATAAGGCTATGAAGAGATACGCCCTGGTTCCTGGAACAATTGCTTTTACAGATGCACATATCGAGG
TGGACATCACTTACGCTGAGTACTTCGAAATGTCCGTTCGGTTGGCAGAAGCTATGAAACGATATGGGCTGA
ATACAAATCACAGAATCGTCGTATGCAGTGAAAACTCTCTTCAATTCTTTATGCCGGTGTTGGGCGCGTTATT
TATCGGAGTTGCAGTTGCGCCCGCGAACGACATTTATAATGAACGTGAATTGCTCAACAGTATGGGCATTTC
GCAGCCTACCGTGGTGTTCGTTTCCAAAAAGGGGTTGCAAAAAATTTTGAACGTGCAAAAAAAGCTCCCAAT
CATCCAAAAAATTATTATCATGGATTCTAAAACGGATTACCAGGGATTTCAGTCGATGTACACGTTCGTCAC
ATCTCATCTACCTCCCGGTTTTAATGAATACGATTTTGTGCCAGAGTCCTTCGATAGGGACAAGACAATTGCA
CTGATCATGAACTCCTCTGGATCTACTGGTCTGCCTAAAGGTGTCGCTCTGCCTCATAGAACTGCCTGCGTGA
GATTCTCGCATGCCAGAGATCCTATTTTTGGCAATCAAATCATTCCGGATACTGCGATTTTAAGTGTTGTTCCA
TTCCATCACGGTTTTGGAATGTTTACTACACTCGGATATTTGATATGTGGATTTCGAGTCGTCTTAATGTATAG
ATTTGAAGAAGAGCTGTTTCTGAGGAGCCTTCAGGATTACAAGATTCAAAGTGCGCTGCTGGTGCCAACCCT
ATTCTCCTTCTTCGCCAAAAGCACTCTGATTGACAAATACGATTTATCTAATTTACACGAAATTGCTTCTGGTG
GCGCTCCCCTCTCTAAGGAAGTCGGGGAAGCGGTTGCCAAGAGGTTCCATCTGCCAGGTATCAGGCAAGGA
TATGGGCTCACTGAGACTACATCAGCTATTCTGATTACACCCGAGGGGGATGATAAACCGGGCGCGGTCGGT
AAAGTTGTTCCATTTTTTGAAGCGAAGGTTGTGGATCTGGATACCGGGAAAACGCTGGGCGTTAATCAAAGA
GGCGAACTGTGTGTGAGAGGTCCTATGATTATGTCCGGTTATGTAAACAATCCGGAAGCGACCAACGCCTTG
ATTGACAAGGATGGATGGCTACATTCTGGAGACATAGCTTACTGGGACGAAGACGAACACTTCTTCATCGTT
GACCGCCTGAAGTCTCTGATTAAGTACAAAGGCTATCAGGTGGCTCCCGCTGAATTGGAATCCATCTTGCTC
CAACACCCCAACATCTTCGACGCAGGTGTCGCAGGTCTTCCCGACGATGACGCCGGTGAACTTCCCGCCGCC
GTTGTTGTTTTGGAGCACGGAAAGACGATGACGGAAAAAGAGATCGTGGATTACGTCGCCAGTCAAGTAAC
AACCGCGAAAAAGTTGCGCGGAGGAGTTGTGTTTGTGGACGAAGTACCGAAAGGTCTTACCGGAAAACTCG
ACGCAAGAAAAATCAGAGAGATCCTCATAAAGGCCAAGAAGGGCGGAAAGATCGCCGTGTAAT

Color codes: yellow, green and gray as in Scheme S1; red, sites of single nucleotide mutation G to A.

Table S1. Primers used for qRT-PCR and ChIP.

Sequence	Name	Method
For ACTGGAGACAAAGTGGGAGCC<br>Rev CAGACACTGGCAACATTGCG	RPLP0_mRNA	qRT-PCR
For CACCGAGTCGTAGTCGAGGT<br>Rev TTTCGGGTAGTGGAAAACCA	Myc_mRNA	qRT-PCR
For CGAGGTAGGCGTGTACGGTG<br>Rev CAGGAGGCTGGATCGGTCC	Luc_promoter	qRT-PCR<br>/ChIP
For GCACATATCGAGGTGGACATCA<br>Rev TCAGCCCATATCGTTTCATAGC	Luc+0.2	qRT-PCR<br>/ChIP
For TCAACAGTATGGGCATTTCGC<br>Rev GTGACGAACGTGTACATCGACTG	Luc+0.4	qRT-PCR<br>/ChIP
For CAACCCTATTCTCCTTCTTCGC<br>Rev GATACCTGGCAGATGGAACCTC	Luc+1	qRT-PCR<br>/ChIP
For TTCTCAGAGGCTTGGCGGGA<br>Rev GCAGCTCTGCTCGCCCCG	MYC(h)_ChIP+0.1	ChIP
For TGGAGAGGGAAGGTTGGGAG<br>Rev TGAAGGAGAAGGCGAGAGGC	Myc_ChIP+1.5	ChIP
For CGAGGAGAATGTCAAGAGGCG<br>Rev GGACAGGATGTATGCTGTGGC	Myc_ChIP+4.85	ChIP
For CTGCTTGCCGCCCTCTTTGG<br>Rev CACAGCCAGACGGACAATGAG	SLC38A2_mRNA	qRT-PCR
For CGTATCCGCATCCACATCCAC<br>Rev TGCTCGTCCCACTTCCTCAAG	PHLDA1_mRNA	qRT-PCR
For TCATACTCGGGCAAGGCAGC<br>Rev GAGGCAGCGAACCAGACAGC	TRIB3_mRNA	qRT-PCR
For CAGTCGTCCTTGCTGGATGTG<br>Rev CCAGGAGAAACTGCCACAGC	ETS2_mRNA	qRT-PCR
For TTAACCACAACCCCGACGC<br>Rev CCCGTGTTTTCCTGCCGTAA	LHX2_mRNA	qRT-PCR
For GAGCTTTTGGTGTCCTTGGGT<br>Rev CTCTCTGGCAGCATCTGAAGG	NFkBIA_mRNA	qRT-PCR
For TGAGCCGCGACTGTGATG<br>Rev GTCTCGGTGACAAAGTCGAAGTT	P21_mRNA	qRT-PCR/<br>ChIP
For AAAACTCGACGCAGAGCTGTG<br>Rev AGGTGAGAAGCAGGTTGTGGAC	ARL6IP1_mRNA	qRT-PCR
For GGCTTCCACACAACTCCAGTG<br>Rev CCGCTGTAGGTTTCATGGTGG	TMEM123_mRNA	qRT-PCR
For CGGTGGTCTCTATTCTCACGCAG<br>Rev TTCCCTTGACACGGCGGACG	SLC38A2_ChIP_TSS	ChIP
For CTTCAATGTTCGTTAATGCCTGG<br>Rev GCACAGAATGGTGACACATGCG	SLC38A2_ChIP_CDS	ChIP
For GCAGCCGCTACTCTCCAGCATC<br>Rev TCTGCCTCCTGCGTGTTAGCC	PHLDA1_ChIP_TSS	ChIP
For GAGGCTGAGACAGGAGAATGGC<br>Rev GGCTCCCTCTTGGTGGCTGG	PHLDA1_ChIP_CDS	ChIP
For GTGTTACACGCACGCACGCC<br>Rev CCGCACGCCCTCCTCTAGG	TRIB3_ChIP_TSS	ChIP
For GAAGCGGTTGGAGTTGGATGAC<br>Rev CGGGCTCCAGGAGGACATAGG	TRIB3_ChIP_CDS	ChIP
For GCGATCAGCACCACGACTCG	ETS2_ChIP_TSS	ChIP

Rev GTGGCACCGAAGGGCAGCCG		
For GCCGTCCGATTGTTCTGTTCC<br>Rev TGCTGCCTGGGAAGTCCTGACT	ETS2_ChIP_CDS	ChIP
For GGAGAAACCCCTACTCCAGTCG<br>Rev GTTCACCCTGAAGCCGCTAGG	LHX2_ChIP_TSS	ChIP
For GCCTCGGAGATGGTGATGCG<br>Rev CGGCATGGTTGAAGTGTGCG	LHX2_ChIP_CDS	ChIP
For CAGAGGACGAAGCCAGTTCTC<br>Rev GCCCTATAAACGCTGGCTGG	NFkBIA_ChIP_TSS	ChIP
For CTCTTCCCCACAGGTTCCTG<br>Rev GCAGGTTGTTCTGGAAGTTGAG	NFkBIA_ChIP_CDS	ChIP
For TAGCCCAGATTCCCTCTCCG<br>Rev GCTCCAAGATGCTTTTCCTCAC	P21_ChIP_TSS	ChIP
For GGGGACAGGCAGCCAGGAC<br>Rev CTCGGGTTGTGGTTGGTGTG	ARL6IP1_ChIP_TSS	ChIP
For CGGACAGAACAGATGGATCTAGA<br>Rev CTCTGAGAGGCGGTGAATAGTTA	ARL6IP1_ChIP_CDS	ChIP
For GCAGCCACTTACCCGCCATG<br>Rev CGAAGGGGAAGCGGAGCCG	TMEM123_ChIP_TSS	ChIP
For GGCACTAGGGCAGGTTATTGG<br>Rev CTGGGCTTCCACACAACTCC	TMEM123_ChIP_CDS	ChIP
